# Age-Dependent Causes of Death among Patients with Breast Cancer Based on Osaka Cancer Registry and Vital Statistics in Japan

**DOI:** 10.3390/healthcare11101409

**Published:** 2023-05-12

**Authors:** Mayumi Nagayasu, Toshitaka Morishima, Makoto Fujii, Haruka Kudo, Tomotaka Sobue, Yuko Ohno, Isao Miyashiro

**Affiliations:** 1 Division of Health Sciences, Graduate School of Medicine, Osaka University, 1-7 Yamadaoka, Suita 5650871, Japan; 2 Department of Nursing, Hyogo Medical University, 1-3-6 Minatojima, Kobe 6508530, Japan; 3 Cancer Control Center, Osaka International Cancer Institute, 3-1-69 Otemae, Osaka 5418567, Japan; 4 Division of Environmental Medicine and Population Sciences, Department of Social and Environmental Medicine, Graduate School of Medicine, Osaka University, 2-2 Yamadaoka, Suita 5650871, Japan; 5 Institute for Cancer Control, National Cancer Center Japan, 5-1-1 Tsukiji, Tokyo 1040045, Japan

**Keywords:** breast cancer, causes of death, cancer registry, mortality hazard

## Abstract

We aimed to clarify the differences in causes of death among patients with breast cancer according to age at diagnosis and years elapsed since diagnosis. Using data from the Osaka Cancer Registry and Vital Statistics databases, 40,690 female patients diagnosed with primary breast cancer between 1985 and 2006 were included in this study. The statistics on all deaths between 1985 to 2016 were collected, and the observation period was 10 years (2006–2016). Mortality hazards according to age at diagnosis and years elapsed since diagnosis were estimated using a flexible parametric estimation. Of the 40,690 patients, 13,676 (34%) died from all-cause death, and the 10-year survival rate was 65.74% (95% confidence interval: 65.28–66.21). The proportions of deaths were 10,531 (77%) from breast cancer, 1048 (8%) from other cancers, and 2097 (15%) from non-cancer causes. The mortality hazard for deaths from breast cancer was initially high and then declined, whereas that for deaths from other cancers and non-cancer causes was initially low and then increased. The more likely causes of death 5 years after breast cancer diagnosis were other cancers or non-cancer causes among patients aged ≥70 years.

## 1. Introduction

Cancer has been the leading cause of death in Japan since 1981 [1]. In Japan, 381,505 people died of cancer in 2021, accounting for 26.5% of all deaths [2]. The age-standardized mortality rate from all causes at all sites for women was 57.7 in 2021. Age-standardized mortality rates for cancer increase gradually from the age of 50 years and significantly after the age of 60 years. In 2019, the number of breast cancer cases in Japan was 97,142, making it the most common cancer type among women. The age at which breast cancer commonly occurs is bimodal, between 45–49 and 60–65 years, making it common in a wide range of age groups [3]. In 2021, there were 159,038 deaths from all cancers among women, with breast cancer accounting for 14,803 deaths, i.e., 9.3% of all cancer-related deaths in women; hence, breast cancer is one of the most common cancers in women [4,5].

The possibility of death from primary cancers in years after diagnosis can either be high in the first 5 years after diagnosis and then gradually decline, as in the case of stomach and colon cancers, or remain high from the outset, as in the case of liver cancer. Breast cancer has a high survival rate but is characterized by a constant probability of death 5 years after diagnosis [6,7]. Furthermore, few studies have examined the causes of death among breast cancer survivors in detail in Japan, although some have examined survival and mortality rates without distinguishing the causes of death according to years since diagnosis and others have examined deaths based on clinical stage. In several studies on the cause of deaths among patients with breast cancer, the incidence of the disease was either considered in one group of elderly patients or elderly patients aged ≥75 years were not included [8,9,10,11,12]. Therefore, the present study aimed to characterize the causes of death in patients with breast cancer according to the age at which they were affected. Breast cancer has a stronger social impact than other cancers on patients and their families because it is common in younger age groups with social roles. In addition, delays in treatment behaviours, such as cancer screening and hospital visits, may contribute to difficulties in continuing treatment and early death. Because breast cancer has a high survival rate and long-term survival is possible, it is important to understand the actual situation after a cancer diagnosis.

## 2. Materials and Methods

This was a population-based cohort study. The primary endpoint of this study was the cause of death in patients with breast cancer according to age at diagnosis and years elapsed since diagnosis.

### 2.1. Database

This study extracted data from the Osaka Cancer Registry (OCR) and Vital Statistics databases. The current study was one of several studies performed by Neoplasms ANd other cause of DEath (NANDE), a collaborative research group [13,14,15]. The OCR (covering the Osaka population of 8 million) was used to collect information on cancer incidence, whereas Vital Statistics was used for information on the causes of death [16]. The OCR contains cancer diagnoses and patient characteristics (sex and age at diagnosis). For the classification of cancer sites, the third edition of the International Classification of Diseases for Oncology (ICD-O-3) was used. Causes of death were registered using the ninth edition of the International Classification of Diseases (ICD9) from 1985 to 1994 and the tenth edition of the ICD10 from 1995 onwards. Information from the OCR and Vital Statistics were linked using nine indicators: prefecture of residence at the time of death; city, town, or village of residence at the time of death; sex; year, month, and day of date of birth; year, month, and day of date of death [17]. These data covered 96.5% of all deaths in the OCR. The data generated were anonymized by removing the birth and death dates and the city, town, or village of residence after linking.

Information on cancer incidence was based on the data of 1,063,987 people from the OCR between 1985 and 2013. Information on the causes of death was based on 32,144,355 death certificates from Vital Statistics for all deaths between 1985 and 2016.

### 2.2. Selection of Analysis Participants and Period

The participants included in the analysis were patients with primary breast cancer (ICD-O-3: C50) in the OCR who were diagnosed between 1985 and 2006. The exclusion criteria were as follows: cancer death certificate only (DCO), diagnosis of epithelial cancer with a good prognosis, and 0 days alive or unknown last month of confirmed survival. Of the 48,890 patients with primary breast cancers, 3199 males and 1 person aged ≤14 years with childhood cancer were excluded; thus, 40,690 participants were included in the final analysis. The observation period was set at 10 years.

### 2.3. Statistical Analyses

This study assessed the changes in the mortality hazard over time according to age at breast cancer diagnosis and the cause of death after diagnosis. Information on the cause of death was obtained for deaths occurring between 1985 and 2016. The causes of death were classified into four categories: all-cause death, death from breast cancer (ICD9: 174, ICD10: C50), death from cancers other than breast cancer (ICD9: 140–239 or ICD10: C00–96), and non-cancer causes (all codes except deaths from breast cancer and deaths from cancers other than breast cancer). The age at diagnosis was classified into 14 age groups at 5-year intervals: 15–29, 30–34, 35–39, 40–44, 45–49, 50–54, 55–59, 60–64, 65–69, 70–74, 75–79, 80–84, 85–89, and ≥90 years. Those aged 15–29 and ≥90 years were classified into one group because of their small numbers. The results are tabulated according to the 14 categories.

The 10-year crude observed survival rate was calculated using the Kaplan–Meier method and corrected survival rates were calculated by treating the other cause of deaths occurring with 5 or 10 years of follow-up as withdrawals. For example, when calculating the adjusted survival rate for breast cancer deaths, other cancer deaths and non-cancer deaths were considered censored. The 10-year corrected survival rate of 50% for breast cancer indicates that 50% of breast cancer patients are spared the risk of death from the disease within 10 years of diagnosis [18].

The four causes of death and censoring were examined descriptively over 10 years after a breast cancer diagnosis, and the changes were compared according to the age group at diagnosis. The Royston–Parmar model of the flexible parametric survival models was used to estimate the mortality hazard according to the cause of death in the years since cancer diagnosis. This hazard model can estimate the mortality hazard at all time points after breast cancer diagnosis and can be visualized smoothly over time [19,20]. In the present study, each cause of death from morbidity was compared by illustrating the relationship between mortality hazard and time. Finally, the detailed cause-of-death distribution for each of the three age groups, 15–44, 45–69, and ≥70 years, is summarized in terms of number and percentage. Statistical significance was set at *p* = 0.05, and all analyses were performed using STATA ver. 15 (Stata Corp., College Station, TX, USA).

### 2.4. Ethics Approval

The need for patient consent was waived because the information from the OCR was processed in accordance with the Cancer Registry Promotion Act to obtain all data without personally identifiable information. In addition, anonymized information from the Vital Statistics was obtained according to the Static Act. This study was approved by the Ethical Review Committee of the Osaka International Cancer Institute (approval number: 1707105103).

## 3. Results

Of the 40,690 patients with breast cancer, 13,676 (34%) died from all-cause death. The 10-year survival rate was 65.74% (95% confidence interval: 65.28–66.21). For patients aged ≥70 years, the 10-year survival rate for localized disease was 60.13% (58.44–61.78), which was lower than that for patients aged 15–44 years and 45–69 years. The 10-year corrected survival rates for breast cancer deaths were 90.95% (90.53–91.36) for localized stage, 65.60% (64.76–66.43) for regional, and 12.04% (10.63–13.54) for distant disease, but did not differ significantly by age group. The 10-year corrected survival rates for non-cancer deaths were in the 90% range for those aged 69 years and younger and ranged from 64.67% to 73.36% for those aged ≥70 years (Table 1).

The proportions deaths according to the causes of death were 10,531 (77%) from breast cancer, 1048 (8%) from other cancers, and 2097 (15%) from non-cancer causes.

Regarding the proportions for each cause of death according to the number of deaths in each 5-year age group of patients with breast cancer, the deaths from breast cancer decreased with increasing age at diagnosis. Deaths from breast cancer accounted for approximately ≥80% of the deaths in patients aged ≤64 years; however, there was a significant decline in deaths from breast cancer among those aged ≥65 years. The proportion of deaths from breast cancer decreased to 48% and 37–42% in the 75–79 and >80 years age groups, respectively. The proportion of deaths from other cancers increased with increasing age at diagnosis, with a range of 0–8% for patients aged ≤64 years, 10–14% for those aged 65–89 years, and 6% for those aged ≥90 years. The proportion of deaths from non-cancer causes gradually increased with increasing age at diagnosis, with a range of 1–6% for patients aged ≤59 years and increased significantly for those aged >60 years. Death from non-cancer causes accounted for more than half of all deaths among patients aged ≥80 years (Table 2).

**Figure 1:**
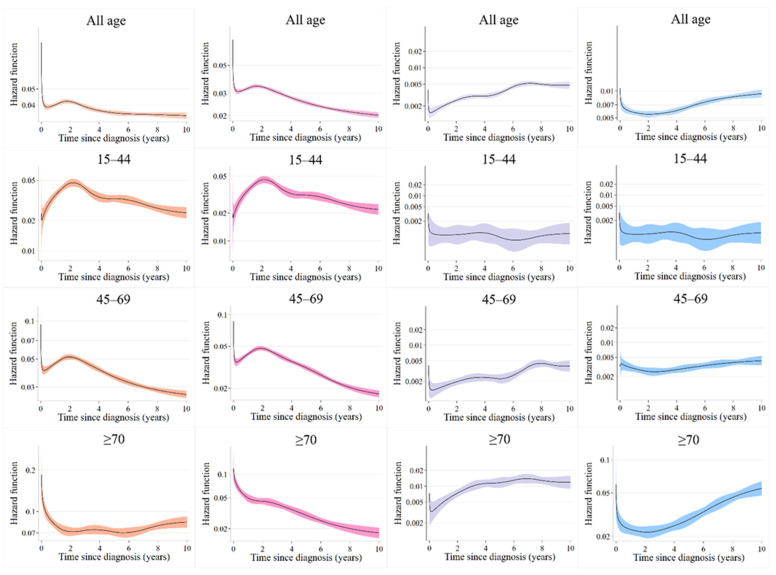
Mortality hazard during the 10-year period after diagnosis by age group.

**Figure A1:**
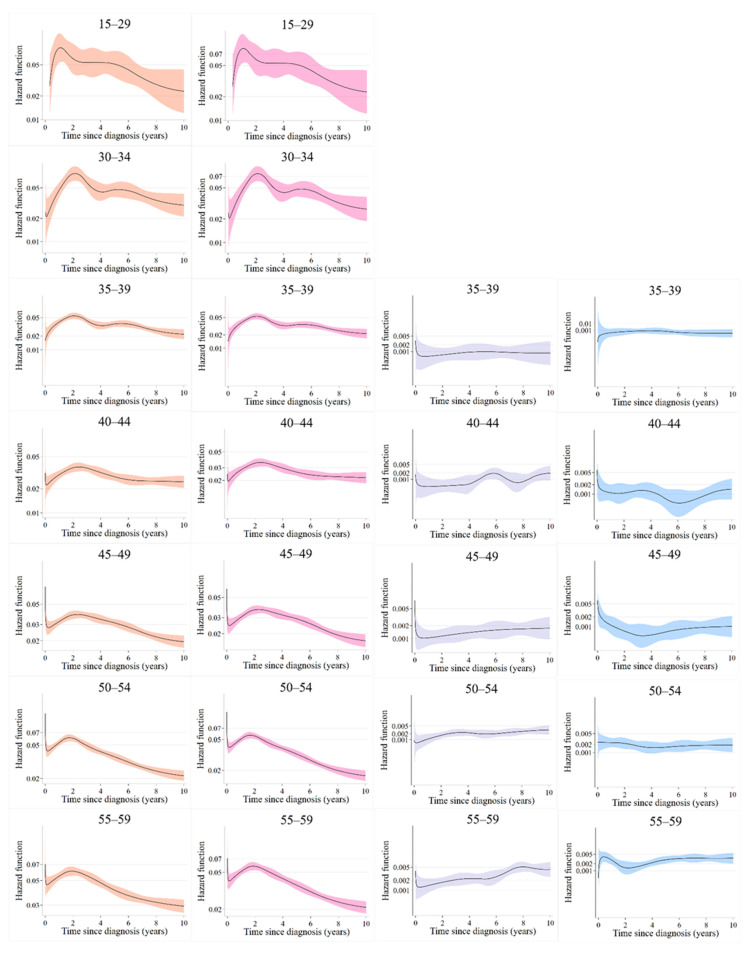

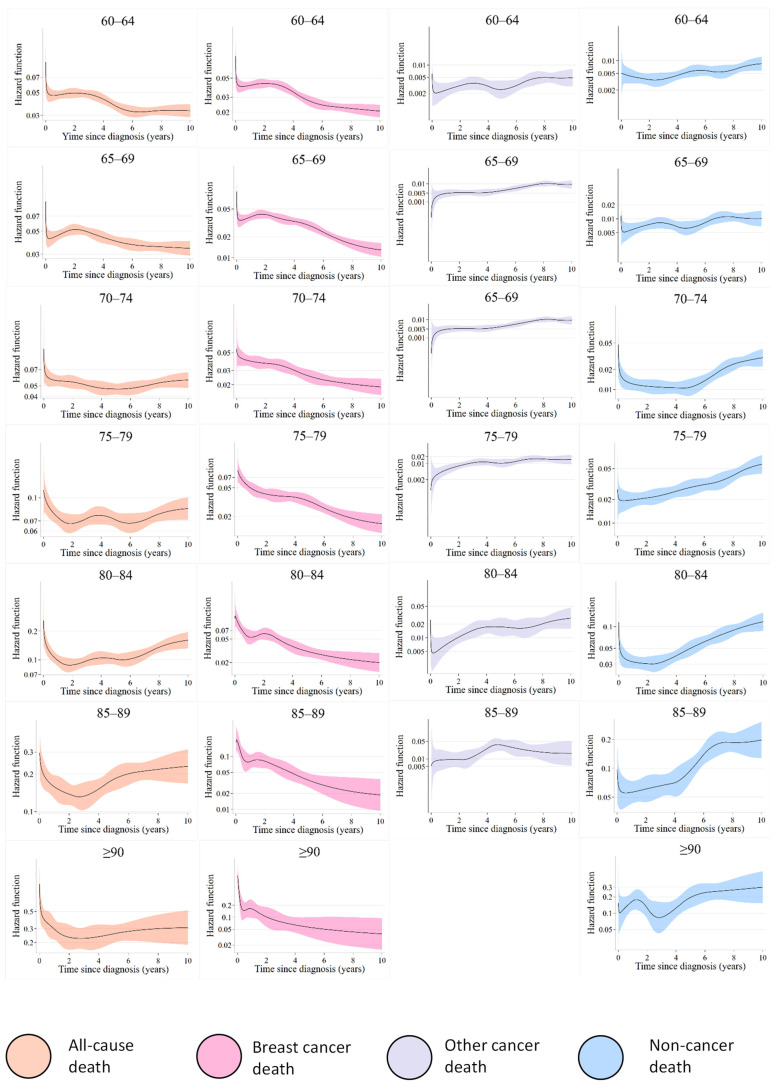
Mortality hazard during the 10-year period after diagnosis.

For age groups (Figure 1), the mortality hazard for all-cause death increased after the breast cancer diagnosis, peaked in the second year, declined until the fifth year, and then flattened after the fifth year. The mortality hazard for death from breast cancer peaked in the second year after diagnosis and then showed a downward trend, gradually declining until the 10th year. The mortality hazard for death from other cancers increased gradually until the sixth year after diagnosis and remained almost constant from the seventh year onwards. The mortality hazard for death from non-cancer causes remained low initially after diagnosis but gradually increased with years that elapsed until the 10th year. In the breast cancer death group, the mortality hazard according to age at diagnosis showed a decreasing trend from age 15 to 69 years, peaking around the second year and declining later. On the other hand, the trends of death from breast cancer for those aged above 70 were high at the beginning but declined 5 years after diagnosis. There was an increasing trend in the mortality hazard for death from other cancers, but the increase was not significant in any age group; however, the value of the mortality hazard was higher for those aged ≥60. The mortality hazard for deaths from non-cancer causes was flat for patients aged 15–59 years, but there was a significant increase with the years elapsed since diagnosis for those aged ≥60 years. The value of the mortality hazard for those aged ≥70 years was high and increased markedly after 5 years from diagnosis for those aged 70–74 and continued to rise from the beginning for those aged ≥75 (Figure A1). The hazards could not be calculated for deaths from other cancers and non-cancer causes in the 15–29 and 30–34-year age groups and death from other cancers in the group aged >90 years due to the small number of participants.

**Figure 2:**
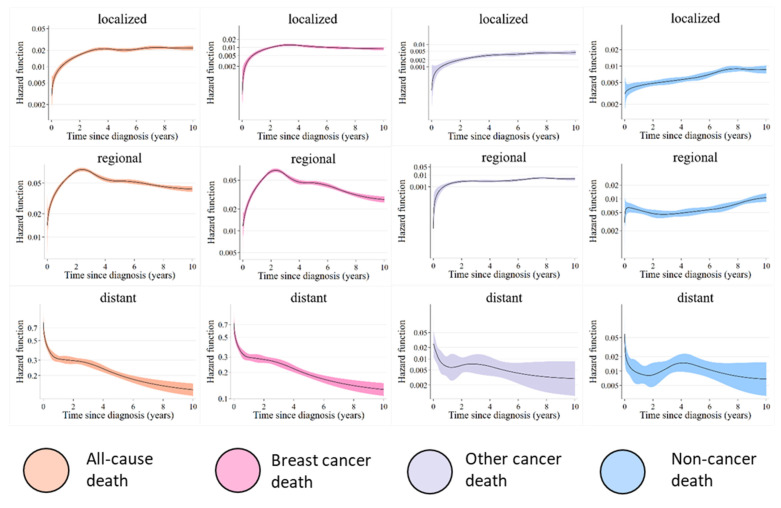
Mortality hazard during the 10-year period after diagnosis by stage.

Based on these results, we summarized age groups with similar mortality hazard trends and reclassified breast cancer patients into three age groups (15–44, 45–69, and ≥70 years) to estimate mortality hazards. In the breast cancer death group, the mortality hazard according to age at diagnosis showed a peak around the second year and later decreased for deaths from all age groups. The mortality hazard for deaths from non-cancer causes was flat for patients aged 15–69 years, but there was a significant increase with the years elapsed since diagnosis for those aged ≥70 years (Figure 1). The results were also examined by clinical stage, but no significant differences by age were observed (Figure 2).

A summary of the detailed distribution of causes of death is shown in Table 3. In all, 94.98% of deaths in the group aged 15–44 years were due to breast cancer; the same was true for the group aged 45–69 years, where 84.76% of deaths were due to breast cancer; for the group aged ≥70 years, the percentage of breast cancer deaths was 47.4%, which was lower than for the other two groups. For those aged ≥70 years, the causes of death were major cancers such as stomach cancer, colorectal cancer, and lung cancer. Regarding non-cancer causes, cardiovascular diseases accounted for the highest percentage at 18.84%, followed by respiratory diseases at 8.96%.

## 4. Discussion

In this study, the change in mortality hazard among patients with primary breast cancer was determined according to the cause of death. The OCR, from which the information on cancer incidence in this study was derived, was initiated in 1962, and it is one of the largest regional cancer registries, with a sample of >8 million people [21]. The accuracy of the OCR is recognized internationally; it is an observer in the CONCORD study, an international collaborative cancer survival study, and it is included in Cancer Incidence in Five Continents (CI5) which is published by the International Association of Cancer Registries, an international organization [22]. Vital statistics provide the oldest and most accurate information on the causes of death in Japan [23]. These statistics are based on the death certificates of deceased residents. One cause of death was identified for each patient. Since this study linked information from the OCR database, where all cases are registered, with that in the Vital Statistics database, the results are considered highly reliable.

A national cancer registry was launched in Japan in January 2016. Considering the long-term prognostic tracking and accuracy of the source registries, this study’s findings provide important information for breast cancer survivors. Japan’s healthcare system is a universal health insurance system, with a high-cost medical expense benefit system that reduces out-of-pocket costs. In addition, it includes a free-access system that allows patients to choose medical institutions freely [24]. This medical system allows for aggressive treatment at any age in Japan and is considered the standard of care. There are guidelines for breast cancer treatment from the Japanese Breast Cancer Society, which are revised every 2–3 years, and breast cancer is one of the cancer types for which standard treatment has long been established [25]. Therefore, patients with breast cancer vary according to the stage, background, and treatment centre, although most patients are considered to receive standard treatment according to the guidelines. The mortality hazard for all-cause deaths in patients with breast cancer peaked in the second year, declined until the fifth year, and then plateaued after the fifth year. This indicates a risk of death after the fifth year after breast cancer diagnosis. In previous studies, exponential models rather than Weibull models have commonly been used to estimate breast cancer survival functions. However, the analysis of mortality hazards according to the cause of death in the present study showed that deaths from breast cancer were characterized differently, with a high and then gradually declining mortality hazard in the early years after diagnosis, whereas deaths from other cancers and non-cancer causes had a low and then gradually increasing mortality hazard in the early years after diagnosis. Previous findings of mortality after the fifth year of a breast cancer diagnosis can be attributed to the combined effect of deaths from other cancers and non-cancer causes rather than deaths from breast cancer [6,7]. Examination of breast cancer diagnosis according to age group showed a decrease in the mortality hazard for death from breast cancer after the fifth year of diagnosis in all age groups and an increasing trend in the mortality hazard for deaths from other cancers; however, the increase was not significant in any age group. The mortality hazard for deaths from non-cancer causes was flat for patients aged 15–69 years, whereas there was a significant increase in the years elapsed since diagnosis for those aged ≥70 years. Based on these results, a certain number of deaths were still observed after 5 years after breast cancer diagnosis, possibly because of deaths from other cancers and non-cancer causes at the age of ≥70 years. Patients aged 75 years or older were included in this study. The other cancer deaths were caused by the most commonly occurring cancers such as colorectal cancer and gastric cancer. We examined deaths from non-cancer causes in detail in the present study; deaths due to circulatory and respiratory diseases were particularly common among patients with breast cancer aged ≥70 years. It is possible that age-dependent causes of death, as described in previous studies, may be a greater risk than death from breast cancer, particularly in older patients with breast cancer. Previous Swedish studies [26,27] have also reported an increase in deaths from other cancers and cardiovascular diseases in older patients aged 65–74 years at the time of breast cancer diagnosis, consistent with our results. The present study included patients aged ≥75 years, and this may have had implications in terms of deaths from other cancers and non-cancer causes.

The trend of deaths from breast cancer deaths continually declining after a peak in the second year in all age groups indicated that patients with breast cancer have a good prognosis. The relative survival rates (1, 3, 5, and 10 years) according to clinical progression from 1993 to 2006 were >90% for all localized breast cancers and 98.8%, 90.4%, 81.9%, and 68.3% for regional breast cancers, respectively. In contrast, more than half of patients with distant breast cancer have been reported to die by the third year after diagnosis: 75.5%, 44.0%, 28.4%, and 14.7%, respectively [28]. These rates are consistent with the high rate of deaths from breast cancer in the second year.

In Japan, international cause-of-death selection rules have been adopted for causes of death, and death from breast cancer is likely to be selected as the primary cause of death [29]. However, the present study’s results showed that patients with primary breast cancer died from causes other than breast cancer. Even during the relatively short observation period of 10 years, there were cases of deaths from other cancers and non-cancer causes, possibly because breast cancer was not mentioned on the death certificate. This suggests that breast cancer may not directly cause death and that the risk of dying from breast cancer may be lower than that currently reported and depends on the age at diagnosis and years elapsed since diagnosis [30]. In addition, the 10-year mortality rate for patients with breast cancer aged ≤59 years was 25–35%, which was lower than the 52–94% rate for those aged ≥75 years. Breast cancer deaths account for >80% of deaths among those aged ≤59 years or younger; therefore, it is important to support early detection and treatment of breast cancer in this age group.

Breast cancer has a greater social impact on patients and their families because it is more common in younger age groups with social roles than other cancers. In addition, delays in treatment behaviours, such as cancer screening and hospital visits, may contribute to difficulties in continuing treatment and early death. In Japan, cancer control measures for breast cancer have included breast cancer screening for early detection since 1987, and since 2004, visual and mammographic examinations have been conducted once every 2 years for women aged ≥40 years [31]. According to a report by the National Cancer Center, the breast cancer screening uptake rate among individuals aged 40–69 years was 39.1% in 2010; however, it increased each year and reached 47.4% in 2019 [32]. The mammography screening uptake rate in Western countries exceeds 70% of the eligible population, whereas the overall screening uptake rate in Japan is approximately 40%, which is very low [33]. According to a report by the Organisation for Economic Co-operation and Development, due to the impact of the COVID-19 pandemic, the number of breast cancer screening visits fell by an average of 9% in 2020 compared with 2019 [34]. As a similar trend is expected in Japan, there is an urgent need to improve the breast cancer screening uptake rate to improve early detection and treatment. Support for the continuation of treatment by providing psychosocial support to female patients with a social life, additional testing for hereditary breast cancer, and expansion of additional testing for high-density breasts are also considered necessary measures [35,36]. The number of patients with breast cancer aged <39 years who are not subject to breast cancer screening is not high; however, support is considered necessary because deaths from breast cancer account for >90% of all deaths among patients with breast cancer. It is important to raise awareness of breast cancer and ensure women are monitoring their breasts regularly [37].

This study had several limitations. First, it was difficult to assess the risk of recurrence because it was impossible to discriminate the information on the primary cause of death, i.e., whether death from breast cancer was due to an initial diagnosis or a recurrence. Second, the lack of detailed information on cancer types and treatment did not allow for an examination of the impact of treatment effects. Finally, we could not comparatively analyse the characteristics of death from breast cancer and from other cancers because we were not able to examine the information for cancers other than breast cancer. However, the present study’s results suggest that the causes of death among patients with breast cancer differ according to age at diagnosis and the years elapsed since diagnosis. The assessment of cancer-related mortality at all ages may be valid for primary cancers. Due to the wide range of ages affected by breast cancer and its long-term survivability, an all-age mortality assessment alone is insufficient when considering support for breast cancer survivors. This study’s results on causes of death among patients with breast cancer according to age at diagnosis provide important information for developing treatment strategies against breast cancer. Determining the distribution of the causes of death could be useful for follow-up care and for controlling some specific side effects of therapies such as radiotherapy. They may also contribute to breast cancer care and support strategies for breast cancer survivors.

## 5. Conclusions

We clarified that the causes of death according to age of breast cancer incidence and number of years elapsed since the diagnosis of breast cancer. The fact that a certain number of deaths were still observed 5 years after the diagnosis of breast cancer can be attributed to other cancers in the younger age group and non-cancer causes in older age groups. Non-cancer deaths among those aged 70 years and older included age-related diseases such as cardiovascular and respiratory diseases. The prognosis of breast cancer, which has a high survival rate and affects a wide range of age groups, especially in younger age groups, should be examined based on the age of affected patients and years since diagnosis.

## Figures and Tables

**Table 1:** Summary of cancer incidence and 10-year cancer mortality by cause of death (female, incidence: 1985–2006, death or survival: 1985–2016).

Groups		Incidence	All Cause		Breast Cancer Death		Other Cancer Death		Non-Cancer Death		Censor
			Death	10-Year Crude Observed Survival Rate	Death	10-Year Corrected Survival Rate	Death	10-Year Corrected Survival Rate	Death	10-Year Corrected Survival Rate	
Age	Stage	*n*	*n*	% (95% CI *)	*n*	% (95% CI)	*n*	% (95% CI)	*n*	% (95% CI)	*n*
All	All	40,690	13,676	65.74 (65.27–66.21)	10,531	72.80 (72.35–73.24)	1048	96.63 (96.42–96.82)	2097	93.46 (93.18–93.73)	27,014
	Localized	19,866	3354	82.56 (82.02–83.09)	1678	90.95 (90.53–91.36)	543	96.90 (96.64–97.15)	1133	93.68 (93.31–94.02)	16,512
	Regional	13,303	5298	59.44 (58.59–60.28)	4354	65.60 (64.76–66.43)	332	96.53 (96.14–96.88)	612	93.88 (93.38–94.34)	8005
	Distant	2159	1931	10.26 (9.01–11.60)	1807	12.04 (10.63–13.54)	48	94.75 (92.68–96.25)	76	90.16 (87.22–92.45)	228
	Unknown	5362	3093	41.77 (40.43–43.09)	2692	47.06 (45.66–48.44)	125	96.20 (95.46–96.82)	276	92.28 (91.32–93.15)	2269
15–44	All	8029	2191	72.12 (71.11–73.10)	2081	73.35 (72.36–74.32)	56	99.12 (98.86–99.32)	54	99.19 (98.94–99.38)	5838
	Localized	3899	399	89.41 (88.38–90.35)	356	90.49 (89.51–91.39)	25	99.28 (98.94–99.52)	18	99.51 (99.22–99.69)	3500
	Regional	2670	937	64.22 (62.35–66.03)	897	65.53 (63.66–67.33)	16	99.20 (98.69–99.51)	24	98.80 (98.20–99.20)	1733
	Distant	317	278	11.89 (8.58–15.77)	270	12.77 (9.27–16.86)	<10	96.49 (90.10–98.78)	<10	96.56 (89.93–98.86)	39
	Unknown	1143	577	48.74 (45.78–51.64)	558	49.91 (46.92–52.82)	11	98.56 (97.36–99.21)	<10	99.10 (98.16–99.56)	566
45–69	All	26,133	8046	68.66 (68.09–69.23)	6820	72.96 (72.41–73.51)	556	97.24 (97.01–97.46)	670	96.77 (96.52–97.01)	18,087
	Localized	12,519	1622	86.63 (86.01–87.23)	1030	91.35 (90.83–91.84)	272	97.57 (97.27–97.84)	320	97.20 (96.88–97.48)	10,897
	Regional	8874	3323	61.88 (60.84–62.89)	2895	66.09 (65.07–67.09)	192	97.03 (96.58–97.42)	236	96.50 (96.02–96.92)	5551
	Distant	1441	1288	10.39 (8.87–12.05)	1222	11.77 (10.10–13.58)	31	94.79 (92.05–96.60)	35	93.34 (90.05–95.57)	153
	Unknown	3299	1813	44.61 (42.89–46.31)	1673	47.80 (46.05–49.53)	61	96.98 (96.10–97.66)	79	96.24 (95.30–97.00)	1486
≥70	All	6528	3439	46.25 (45.02–47.48)	1630	71.56 (70.34–72.73)	436	90.07 (89.13–90.94)	1373	71.78 (70.46–73.06)	3089
	Localized	3448	1333	60.13 (58.44–61.78)	292	90.03 (88.87–91.07)	246	91.05 (89.90–92.07)	795	73.36 (71.72–74.93)	2115
	Regional	1759	1038	40.02 (37.69–42.33)	562	63.14 (60.60–65.57)	124	88.63 (86.52–90.42)	352	71.54 (68.83–74.06)	721
	Distant	401	365	8.48 (5.98–11.52)	315	12.67 (9.20–16.71)	13	92.98 (87.25–96.19)	37	72.54 (62.25–80.46)	36
	Unknown	920	703	22.81 (20.12–25.61)	461	40.17 (36.50–43.80)	53	88.04 (84.31–90.94)	189	64.67 (59.99–68.94)	217

* 95% CI denotes 95% confidence intervals. All survival rates in the table are crude survival rates.

**Table 2:** Causes of death within 10 years after diagnosis in women with breast cancer (diagnosed between 1985 and 2006).

				Cause of Death					
Age (Years)	Total No. of Patients	All-Cause Death		Breast Cancer		Other Cancer		Non-Cancer Death	
	No.	No.	(%)	No.	(%)	No.	(%)	No.	(%)
15–29	330	117	35	115	98	0	0	2	2
30–34	913	323	35	315	98	5	2	3	1
35–39	2345	671	29	645	96	16	2	10	1
40–44	4441	1080	24	1006	93	35	3	39	4
45–49	6419	1594	25	1470	92	76	5	48	3
50–54	6043	1882	31	1696	90	96	5	90	5
55–59	5461	1883	34	1655	88	114	6	114	6
60–64	4573	1478	32	1168	79	124	8	186	13
65–69	3637	1209	33	831	69	146	12	232	19
70–74	2738	1086	40	618	57	145	13	323	30
75–79	1976	1018	52	484	48	142	14	392	39
80–84	1177	788	67	318	40	102	13	368	47
85–89	469	389	93	144	37	38	10	207	53
≥90	168	158	94	66	42	9	6	83	53
Total	40,690	13,676	3	10,531	77	1048	8	2097	15

**Table 3:** Detailed distribution of causes of death by age group.

Cause of Death Classification (ICD-10 Code and ICD-9 Code)	Age Group		
	15–44	45–69	≥70
	*n* (%)	*n* (%)	*n* (%)
Certain infectious and parasitic diseases (A00–B99, 001–139)	<10	36 (0.45)	31 (0.9)
Lip, oral cavity, and pharynx (C00–C14, 140–149)	<10	<10	<10
Oesophagus (C15, 150)	<10	14 (0.17)	<10
Stomach (C16, 151)	<10	76 (0.94)	63 (1.83)
Colon (C18, 153)	<10	38 (0.47)	44 (1.28)
Malignant neoplasm of rectum rectosigmoid junction and anus (C19–C20, 154)	<10	17 (0.21)	15 (0.44)
Liver and intrahepatic bile ducts (C22, 155)	<10	62 (0.77)	48 (1.4)
Gallbladder and other biliary tract (23–C24, 156)	<10	33 (0.41)	26 (0.76)
Pancreas (C25, 157)	<10	56 (0.7)	35 (1.02)
Trachea, bronchus, and lung (C33–C34, 162)	<10	61 (0.76)	68 (1.98)
Skin (C40–C41, 172)	<10	<10	<10
Breast (C50, 174)	2081 (94.98)	6820 (84.76)	1630 (47.4)
Cervix uteri (C53, 180)	<10	<10	<10
Corpus uteri (C54, 182)	<10	22 (0.27)	10 (0.29)
Uterus, part unspecified (C55, 179)	<10	10 (0.12)	<10
Ovary (C56, 183)	<10	32 (0.4)	11 (0.32)
Kidney, urinary tract (C62–C66, C68, 189)	<10	<10	<10
Bladder (C67, 188)	<10	<10	<10
Central nervous system (C71–72, 191–192)	<10	<10	<10
Thyroid (C73, 193)	<10	<10	<10
Malignant lymphomas, Hodgkin lymphomas (C81–C83, 200–201)	<10	14 (0.17)	<10
Multiple myeloma (C90, 203)	<10	<10	<10
Leukaemia (C91–C95, 204–209)	<10	21 (0.26)	<10
Other malignant neoplasms (Codes applicable to malignant neoplasms other than the above)	13 (0.59)	74 (0.92)	59 (1.72)
Neoplasms (D1–D49, 210–239)	<10	17 (0.21)	11 (0.32)
Diseases of the blood and blood-forming organs and certain disorders involving the immune mechanism (D50–D89, 280–289)	<10	<10	<10
Endocrine, nutritional, and metabolic diseases (E00–E89, 240–279)	<10	20 (0.25)	24 (0.7)
Mental and behavioural disorders (F01–F99, 290–319)	<10	<10	13 (0.38)
Diseases of the nervous system (G00–G99, 320–389)	<10	18 (0.22)	20 (0.58)
Diseases of the circulatory system (I00–I59, 390–459)	22 (1)	293 (3.64)	648 (18.84)
Diseases of the respiratory system (J00–J99, 460–519)	<10	77 (0.96)	308 (8.96)
Diseases of the digestive system (K00–K95, 520–579)	<10	68 (0.85)	97 (2.82)
Diseases of the skin and subcutaneous tissue (L00–L99, 680–709)	<10	<10	<10
Diseases of the musculoskeletal system and connective tissue (M00–M99, 710–739)	<10	<10	<10
Diseases of the genitourinary system (N00–N99, 580–629)	<10	18 (0.22)	69 (2.01)
Congenital malformations, deformations, and chromosomal abnormalities (Q00–Q99, 740–759)	<10	<10	<10
Symptoms, signs, and abnormal clinical and laboratory findings, not elsewhere classified (R00–R99, 780–799)	<10	<10	76 (2.21)
External cause of morbidity and mortality (V00–Y99, 800–999)	15 (0.68)	91 (1.13)	50 (1.45)

## Data Availability

Not applicable.
